# Integrated Genome Browser App Store

**DOI:** 10.1093/bioinformatics/btac109

**Published:** 2022-02-18

**Authors:** Sameer Shanbhag, Riddhi Patil, Noor Zahara, Chirag Shetty, Rachel Weidenhammer, Sneha Watharkar, Pranav Tambvekar, Philip P Badzuh, Chester Dias, Narendra Vankayala, Prutha Kulkarni, Charan Vallapureddy, Shamika Kulkarni, Pooja Nikhare, Nowlan H Freese, Ann E Loraine

**Affiliations:** Department of Bioinformatics and Genomics, University of North Carolina at Charlotte, Charlotte, NC 28223, USA; Department of Bioinformatics and Genomics, University of North Carolina at Charlotte, Charlotte, NC 28223, USA; Department of Bioinformatics and Genomics, University of North Carolina at Charlotte, Charlotte, NC 28223, USA; Department of Bioinformatics and Genomics, University of North Carolina at Charlotte, Charlotte, NC 28223, USA; Department of Bioinformatics and Genomics, University of North Carolina at Charlotte, Charlotte, NC 28223, USA; Department of Bioinformatics and Genomics, University of North Carolina at Charlotte, Charlotte, NC 28223, USA; Department of Bioinformatics and Genomics, University of North Carolina at Charlotte, Charlotte, NC 28223, USA; Department of Bioinformatics and Genomics, University of North Carolina at Charlotte, Charlotte, NC 28223, USA; Department of Bioinformatics and Genomics, University of North Carolina at Charlotte, Charlotte, NC 28223, USA; Department of Bioinformatics and Genomics, University of North Carolina at Charlotte, Charlotte, NC 28223, USA; Department of Bioinformatics and Genomics, University of North Carolina at Charlotte, Charlotte, NC 28223, USA; Department of Bioinformatics and Genomics, University of North Carolina at Charlotte, Charlotte, NC 28223, USA; Department of Bioinformatics and Genomics, University of North Carolina at Charlotte, Charlotte, NC 28223, USA; Department of Bioinformatics and Genomics, University of North Carolina at Charlotte, Charlotte, NC 28223, USA; Department of Bioinformatics and Genomics, University of North Carolina at Charlotte, Charlotte, NC 28223, USA; Department of Bioinformatics and Genomics, University of North Carolina at Charlotte, Charlotte, NC 28223, USA

## Abstract

**Summary:**

Rapid progress in genome science requires equally rapid visualization software development so that researchers can better explore and understand novel datasets. To make developing new visualizations faster and easier, we previously re-factored the Integrated Genome Browser (IGB), a desktop Java application with dozens of features, into a pluggable application framework that can accept new functionality as plug-ins, called IGB Apps. However, developers lacked a centralized location for sharing Apps, making it hard to connect with potential users. To fill this gap, we created an App Store for IGB, a user-friendly Web site for developers to release and document Apps, and for users to find them.

**Availability and implementation:**

The IGB App Store is available from https://bioviz.org.

## 1 Introduction

Integrated Genome Browser (IGB) is a fast, flexible, free and open-source genome browser implemented in Java that runs natively on users’ personal computers (Nicol *et al.*, 2009). IGB is best known for its responsiveness and fast animated zooming, which evoke the feeling of effortless flight through a genomic data scene. IGB is also highly interactive, offering many ways to manipulate and transform data, from selecting and inspecting individual data items to performing multi-step visual analytics operations on entire datasets, separated into tracks.

Since its first release in the early 2000s, a small community of developers and users has continuously maintained and improved IGB, transforming it into an extensible, pluggable framework based on OSGi (Freese *et al.*, 2016), an open standard for building modular programs in Java. Using this new framework, we and others have developed plug-ins, called ‘IGB Apps’, that add new visual analytics operations, new menu items or entirely new windows to IGB (Céol *et al.*, 2016, 2020; Céol and Müller, 2015; Mall *et al.*, 2016).

However, until now, developers had to deploy and distribute their Apps themselves. They needed to set up a Web site to host their App and then advertise the URL to potential users, who had to enter the URL manually into IGB. This was inconvenient, reducing enthusiasm for the IGB platform among developers and users alike.

To make developing and releasing IGB Apps easier, we created and deployed an App Store for IGB, a Web site where developers can upload and publicize their Apps and users can find them. In this article, we summarize key features of the App Store, including how the App Store, a Web application, communicates with IGB, a native software that runs outside a Web browser.

## 2 Results

### 2.1 Submitting an App

To submit a new App or release a new version of an existing App, a developer first logs in to the App Store via social sign-in (currently, only social sign-in with Google is supported). Next, the developer uploads their App file, implemented as a java archive (jar) file, called a ‘bundle’ in OSGi parlance. The site unpacks the file and checks for compatibility with IGB, displaying a summary report that invites the developer to either submit the App file as-is or replace it with a new version. If the compatibility check passes, the developer can click a button to submit the App, which deploys the jar file to a private testing site which site managers use to install the App into IGB and try it out, manually checking App code and behavior for bugs or security-related red flags, such as sending data to external sites without a user’s knowledge. Managers then either release the App as-is or contact the developer with feedback if changes are needed. Developers and site managers receive email notifications when Apps are submitted or released for public use. Once an App is released, it receives a dedicated home page, which the developer can edit and customize, adding other users as editors. They can also release new versions without further manual review by site maintainers.

### 2.2 Installing an App

**Fig. 1.**
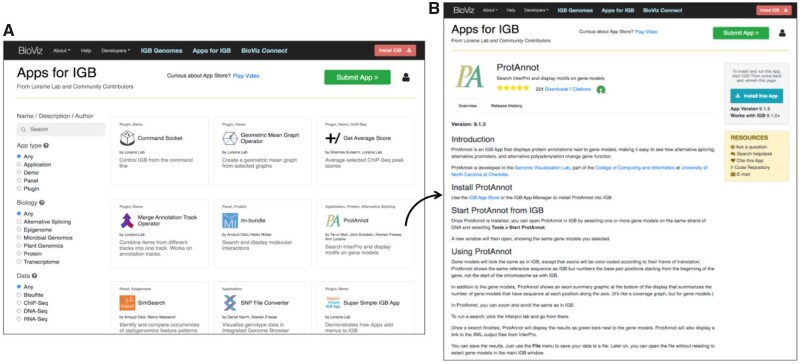
IGB App Store interface. (**A**) App Store home page. (**B**) Home page for ProtAnnot, an IGB App

Finding and installing Apps into IGB is similarly straightforward. The App Store home page shows a grid of App tiles, sorted alphabetically by App name (Fig. 1A), which users can search using a free text query form or a faceted search interface, which filters Apps by categories created and curated by site maintainers. Future App Store releases will likely incorporate categories from systems, such as the EDAM ontology of bioscientific data analysis and data management (Ison *et al.*, 2013), further improving App dissemination and impact.

Clicking an App tile opens the App’s home page, which describes App functionality and allows users to install an App into IGB. If IGB is running, and if the App has not yet been installed, the page displays a button labeled with ‘Install the App’. If the App is already installed, the button displays the text ‘Installed’, and if it is installed but a newer version is available, the button displays the text ‘Update the App’. Once an App is installed or updated, the button label changes to ‘Installed’. If IGB is not running, the button displays a message advising the user to start the IGB program and links to a page where the user can download and install IGB. Users can also install or update Apps within the IGB interface, using the IGB App Manager window, which resembles the App Store interface, making both easier to learn and remember between sessions.

## 3 Materials and methods

We implemented IGB App Store using the Django Web application framework and starting with a fork of the open-source Cytoscape App Store code base (Lotia *et al.*, 2013). We customized and updated the code base extensively, describing two major changes here. First, we re-designed the home page, blending and improving on user interface conventions found across many app store-like applications. For example, we improved on the Grafana Plug-ins Web site faceted search interface by adding tooltips defining the curated categories used. Second, we re-factored the App Store to function as an OSGi Bundle Repository (OBR), conforming to the Apache Felix project OBR specification (Apache Foundation, 2021). On startup, or when a user adds a new App Store, IGB retrieves an OBR-compliant XML document listing the App Store’s available Apps, their versions and their IGB platform requirements, ensuring that IGB installs compatible Apps only. When users visit an App home page, Javascript running in the Web page contacts a localhost REST endpoint within IGB to determine App status, modifying the Web page’s ‘Install this App’ button accordingly (Fig. 1B).

The App Store is open source, and the full commit history of the project is available from https://bitbucket.org/lorainelab/appstore. Ansible playbooks used to deploy and update App Store on Amazon Web Service infrastructure are available from https://bitbucket.org/lorainelab/appstore-playbooks.

Other groups are welcome to deploy their own App Stores, or re-use the code to launch their own projects, with the caveat that supporting an application other than IGB would require modifying the user interface, along with the JavaScript code controlling the App page ‘Install the App’ button, code that depends on interacting with IGB itself via its localhost endpoint.
